# A 3-dimensional finite element analysis to evaluate the impact of force direction, insertion angle, and cortical bone thickness on mini-screw and its surrounding bone

**DOI:** 10.34172/joddd.2021.043

**Published:** 2021-12-05

**Authors:** Omar Nazal Auwer, Marwa Sameh Shamaa, Shaza Mohammad Hammad

**Affiliations:** ^1^Department of Orthodontics, Faculty of Dentistry, Mansoura University, Egypt; ^2^Department of Orthodontics, Faculty of Dentistry, Mansoura University, Mansoura, Egypt

**Keywords:** Cortical bone, Finite element method, Force direction, Insertion angle, Mini-screw

## Abstract

**Background.** The present study aimed to assess the stress and strain distribution on mini-screws and the surrounding bone in cases of different cortical bone thicknesses (CBTs), mini-screw insertion angles, and force directions using finite element analysis (FEA).

**Methods.** Inventor professional version 8 software was used to construct 24 three-dimensional assemblies of mini-screws inserted with different insertion angles (30º, 60º, and 90º) in alveolar bone blocks with different CBTs (0.5, 1, 1.5, and 2 mm). The models simulated mini-screws inserted in bones with different CBTs and different insertion angles. A 2-N load was applied in two directions to mini-screw heads. The resultant stresses of the applied load were collected from the output of the ANSYS program.

**Results.** The results indicated that force direction affected bone strains as the horizontal force generated more strains on cortical bone than the oblique one. Force applied to 60º inserted mini-screws generated much more strains on cortical bone than 90º and 30º inserted mini-screws. In a 60º inserted mini-screw, the horizontal force generated about 45% more strains on cortical bone than the oblique one. The exerted microstrain on bone decreased as CBT increased.

**Conclusion.** It can be concluded that inserting mini-screws at 60º to the bone surface should be avoided as it generates much more strains on cortical bone than 90º and 30º, especially when a force parallel to the bone surface is applied.

## Introduction

Anchorage control plays an important role in orthodontic treatment and significantly affects treatment outcomes as it can minimize undesired movements.^1^ Mini-screws have been extensively applied in orthodontic treatment as a stationary absolute anchorage device because of their various advantages over the traditional methods of skeletal anchorage.^2^

Even so, the clinical behavior of mini-screws is not clear yet. Several authors have reported loosening and failure of mini-screws throughout orthodontic treatment.^3^ Many factors can affect mini-screw stability, including mini-screw type, length, and diameter,^4^ and surface characteristics.^5^ Although different factors might affect mini-screw placement sites such as nearby anatomical landmarks, access, and biomechanics used in treatment, adequate stability is provided when CBT is > 1 mm,^6^ while failure and looseness of orthodontic mini-screws are associated with a thinner cortical bone.^7^

It is impossible to measure stresses accurately on mini-screws and the surrounding bone in vivo. However, most of the suggested ways to increase mini-screw stability and decrease stress concentration on mini-screws and strain concentration on bone were provided without the support of mechanical reasoning. Therefore, no reliable guidelines can be provided for their clinical use without a thorough understanding of the biomechanical rationale of orthodontic mini-screws.

Finite element analysis (FEA) is used to predict the mechanical behavior of different engineering structures^1^ and can be applied to solids of irregular geometries that contain different material properties. FEA has high sensitivity and enables predicting of the stress distribution in the mini-screw‒cortical bone and trabecular bone interfaces, which is a key factor in the success or failure of the mini-screw.^8^

The impact of various insertion angles concerning various force directions on stress and strain distribution on bone and mini-screw is still unknown. Therefore, this study was conducted to investigate the roles of CBT, insertion angle, and force direction in stress distribution on mini-screw and strain distribution in the surrounding cortical bone using FEA.

The null hypothesis was that the relationship between various insertion angles and force directions would significantly affect stress and strain distribution. Also, it was supposed that when CBT is increased, the stress and strain concentration will decrease.

## Methods

### Geometric modeling

**Figure 1:**
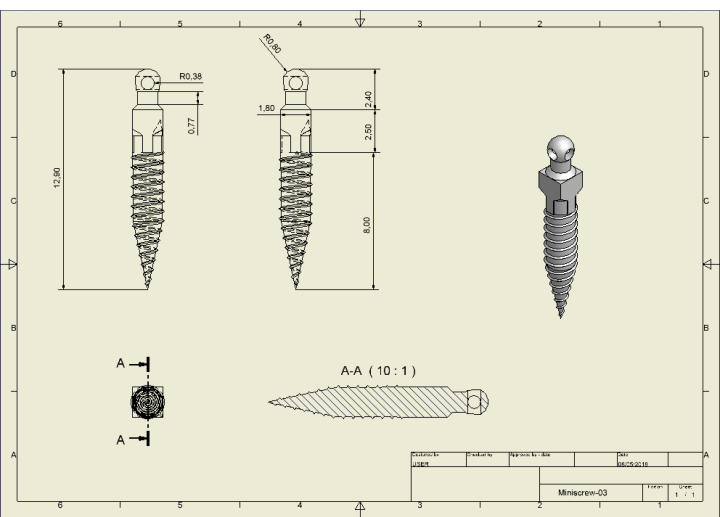
Mini-screw design on Autodesk Inventor screen based on manufacturer dimensions.

Three-dimensional solid modeling software (Autodesk Inventor Professional, Version 8) was used for modeling mini-screws with a diameter of 1.8 mm and a length of 8 mm. The selected mini-screw type was 3M Unitek^TM^ Temporary Anchorage Device (TAD) System (Figure 1) as it provides the following advantages:

1. Multifaceted uses ranging from conventional anchorage to skeletal malocclusions.
2. The threaded body is self-tapping and self-drilling; therefore, there is no need to make a predrilled hole.
3. The apical 4 mm is tapered from 0.3 mm to 1.8 mm. Therefore, the bone is compressed in and around the mini-screw threads rather than cutting and removing bone.

The cortical and spongy bones were modeled in the finite element package. The bone geometry was simplified and simulated as a parallelogram representing cortical bone (20 mm in length × 20 mm in width × 0.5, 1.0, 1.5, and 2 mm in height), the spongy bone (20 mm in length × 20 mm in width × 14.5, 14.0, 13.5, and 13 mm in height). Finally, these components were assembled in an ANSYS environment, and complete osseointegration was assumed.

### Suitable element type selection

Finite element simulations started with selecting the suitable element type according to the structural mass. Several options were available for structure mass types (beam, pipe, shell, and solid). In the present study, the bone model was considered a solid type. Therefore, the element types chosen were tetrahedral and brick.

### Defining the material properties

All the model materials were homogeneous, isotropic, and linearly elastic. The mini-screw was assumed to be pure titanium with a Young’s modulus of 110 GPa and a Poisson’s ratio of 0.35.^9^ For healthy bone quality, the Young’s moduli of the cortical and spongy bones were 14 GPa^9^ and 1.3 GPa,^10^ respectively, and the Poisson’s ratio was 0.3 for both.^9,10^

### Mesh generation

**Figure 2:**
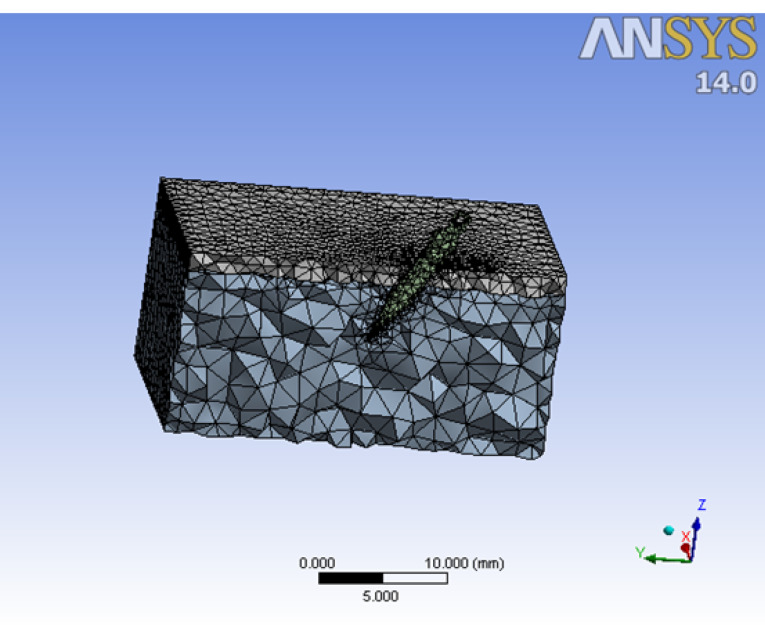
Complete longitudinal cut section in 60º inserted mini-screw.

The accuracy obtained from any FEA model is directly related to the finite element meshing process. In the present study, the mesh generation process involved dividing the previously constructed geometrical model (mini-screw and bone) into small tetrahedral and brick finite elements (Figure 2). The solution functions obtained from these elements were combined to calculate a solution to the whole body. The smaller these elements were made, the more the mesh was refined, and the more accurate the results were.

### Loading and boundary conditions

**Figure 3:**
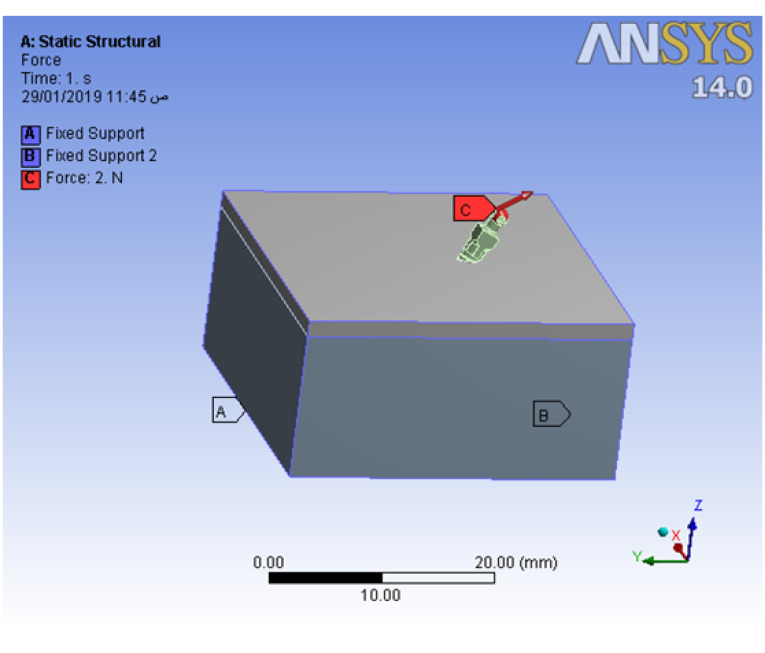
Finite element model showing oblique force applied on 60º inserted mini-screw.

After meshing the model, the next step was to apply structural load and constraints. The restriction of the boundary condition was mandatory to prevent the body from floating, translating, and rotating. The contouring lines of the cortical and spongy bone geometries were set to be fixed as a boundary condition. Then a load of 2 N was applied to the mini-screw head in two directions:

1. Horizontal direction (parallel to the bone surface).
2. Oblique by 30º upward to the horizontal plane (30º upward to the bone surface) (Figure 3).

### Obtaining the solution functions of resultant stresses

A linear static analysis was carried out using a commercial multipurpose finite element software package (ANSYS, Version 14.0). The resultant stresses of the applied load were collected from the output of the ANSYS program, and they were collected in tables and figures according to the maximum values of von Mises stress. In the present work, the results were based on the von Mises stress (Svon) values. To calculate the microstrain in cortical bone, the maximum compressive stress (S3) was used.

This finite element study simulated clinical situations where mini-screws were inserted with various insertion angles (30º, 60º, and 90º) into the bone surface in different cortical bone thicknesses (CBTs) (0.5, 1.0, 1.5, and 2.0 mm), and a 2-N orthodontic force was applied in different directions on all the twelve meshed models.

Statistical significance analyses were not carried out since the results of FEA are individual values without any statistical distribution.^11^

## Results

**Figure 4:**
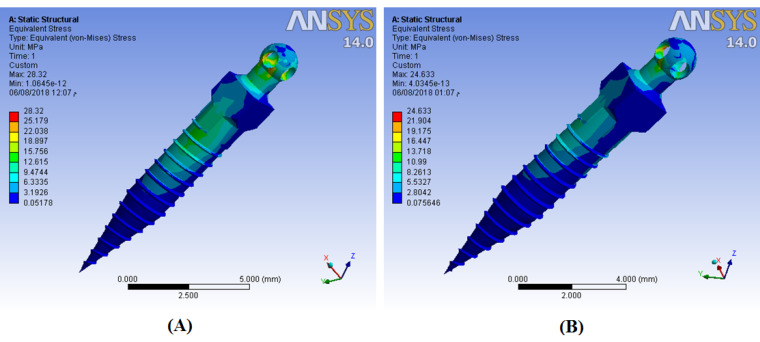
von Mises stress on mini-screws inserted at 60º in bone when CBT was 0.5 mm (A), horizontal force (B), oblique force.

**Figure 5:**
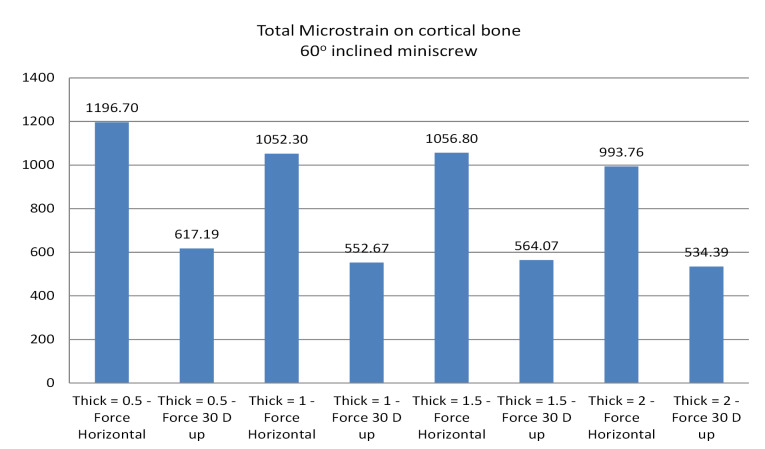
Bar chart showing microstrain on the cortical bone of different thicknesses when insertion angle was 60º to the bone surface and 2-N force applied in two directions.

**Figure 6:**
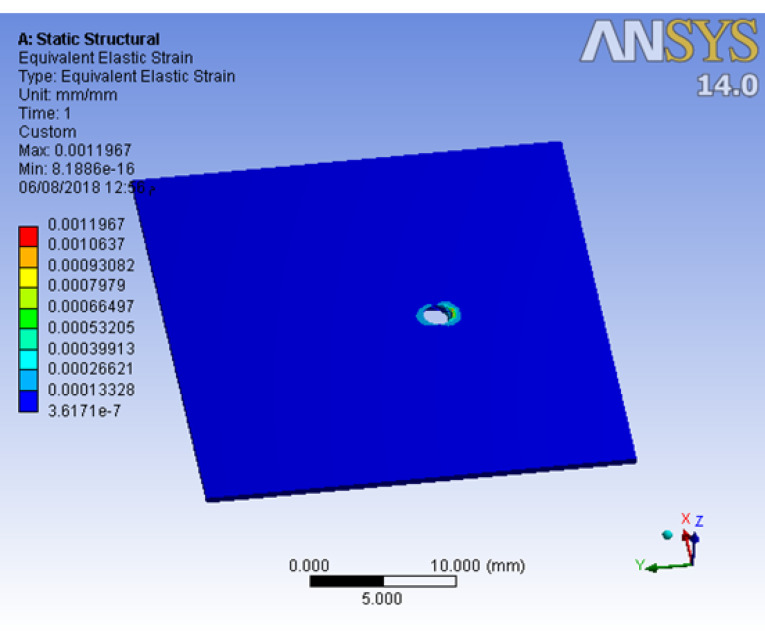
Microstrain on the cortical bone when a horizontal force was applied on 60º inserted mini-screw, and CBT was 0.5 mm.

The results showed that stress distribution on mini-screws and strain distribution on the bone would change if CBT, insertion angle, or force direction change. The horizontal force generated more stresses on the mini-screw body for different CBTs than the oblique one (Figure 4). For all mini-screw insertion angles, mini-screw stresses were insensitive to bone thickness. Mini-screws of 60º insertion angle generated much more strains on cortical bone than 90º and 30º inserted mini-screws. In the 60º insertion angle, the horizontal force generated approximately 45% more microstrains on cortical bone than the oblique one (Figure 5), with the greatest microstrain generated when CBT was 0.5 mm (Figure 6).

Force direction affected bone strains; horizontal force generated more strains on cortical bone than the oblique one. von Mises stresses increased significantly by the direction of forces except for the vertical insertion (Tables 1, 2, 3, and 4). In general, under horizontal force, regardless of the mini-screw angulation, increasing the bone thickness slightly reduced the exerted microstrain on the bone (Figure 5). Under oblique forces and using vertical mini-screws, bone strains were insensitive to CBT.

**Table 1:** Microstrain on the cortical bone of all the modeled insertion angles and force directions with 2-N force when CBT was 0.5 mm

**Insertion angle**	**CBT**	**Force direction**	**Uy**	**Usum**	**Svon**	**Strain Von**
			**Micron**	**Micron**	**MPa**	**Microstrain**
Vertical	0.5	Horizontal	0.348	0.453	6.019	435.83
Vertical	0.5	30 D up	0.302	0.473	5.593	404.82
30 D oblique	0.5	Horizontal	0.000	0.505	6.955	520.94
30 D oblique	0.5	30 D up	0.000	0.169	0.709	506.61
60 D oblique	0.5	Horizontal	0.000	0.591	14.429	1196.70
60 D oblique	0.5	30 D up	0.001	0.282	7.454	617.19

**Table 2:** Microstrain on the cortical bone of all the modeled insertion angles and force directions with 2-N force when CBT was 1.0 mm

**Insertion angle**	**CBT**	**Force direction**	**Uy**	**Usum**	**Svon**	**Strain Von**
			**Micron**	**Micron**	**MPa**	**Microstrain**
Vertical	1.0	Horizontal	0.258	0.357	6.778	489.62
Vertical	1.0	30 D up	0.224	0.385	6.289	454.03
30 D oblique	1.0	Horizontal	0.000	0.356	5.233	494.64
30 D oblique	1.0	30 D up	0.000	0.139	0.567	451.48
60 D oblique	1.0	Horizontal	0.000	0.448	13.416	1052.30
60 D oblique	1.0	30 D up	0.001	0.238	7.007	552.67

**Table 3:** Microstrain on the cortical bone of all the modeled insertion angles and force directions with 2-N force when CBT was 1.5 mm

**Insertion angle**	**CBT**	**Force direction**	**Uy**	**Usum**	**Svon**	**Strain Von**
			**Micron**	**Micron**	**MPa**	**Microstrain**
Vertical	1.5	Horizontal	0.223	0.312	6.761	488.33
Vertical	1.5	30 D up	0.193	0.338	6.254	451.50
30 D oblique	1.5	Horizontal	0.004	0.277	4.216	379.67
30 D oblique	1.5	30 D up	0.000	0.118	0.511	308.70
60 D oblique	1.5	Horizontal	0.004	0.374	13.276	1056.80
60 D oblique	1.5	30 D up	0.001	0.211	7.057	564.07

**Table 4:** The total microstrain on the cortical bone of all the modeled insertion angles and force directions with 2-N force when CBT was 2.0 mm

**Insertion angle**	**CBT**	**Force direction**	**Uy**	**Usum**	**Svon**	**Strain Von**
			**Micron**	**Micron**	**MPa**	**Microstrain**
Vertical	2.0	Horizontal	0.203	0.285	6.613	478.63
Vertical	2.0	30 D up	0.176	0.304	6.099	440.63
30 D oblique	2.0	Horizontal	0.008	0.233	4.342	431.16
30 D oblique	2.0	30 D up	0.000	0.101	0.474	353.30
60 D oblique	2.0	Horizontal	0.017	0.329	12.917	993.76
60 D oblique	2.0	30 D up	0.001	0.190	6.951	534.39

The maximum von Mises stresses generated in the mini-screws and cortical bone were below the yield stress of pure titanium and cortical bone. Therefore, the mini-screws and cortical bone had sufficient strength to withstand force magnitudes up to 2 N. Also, the maximum value of calculated microstrain on the cortical bone was well below the physiologic limit of bone integrity (200 MPa).^11^

## Discussion

Recently, mini-screws have been implemented in most orthodontic treatments, and their success is influenced by mini-screw insertion angle, force direction, and CBT, in addition to other factors.^12^ To maximize the benefits of mini-screws, understanding its mechanical variables is necessary. However, it is impossible to detect the underlying biomechanical mechanisms of mini-screws within the clinical environment due to the restricted mechanical indices that can be measured and inaccurate parameter control. Hence, FEA could be considered a suitable method to estimate stresses and deformations in orthodontic mini-screws simulating real clinical situations.^13^

The results of this study revealed that increasing the CBT slightly reduced the exerted microstrains on bone except when applying oblique force (>30º) on vertically inserted mini-screws in which bone strains were insensitive to CBT. This supported the outcomes of other studies, which found that CBT is directly proportional to the mini-screw success rate. A higher mini-screw success rate was accompanied by a CBT of >1 mm.^14,15^

Our findings were also consistent with Okumura et al,^16^ who found that stresses increased with decreased CBT. They concluded that from a biomechanical point of view, to enhance mini-screw success rates in the posterior maxillary segment, accurate preoperative assessment of the cortical bone at the mini-screw placement location is necessary. Other studies, however, found that CBT did not influence the stresses concentrated in the cortical bone surrounding the mini-screw, which might be due to differences in mini-screw design, geometry, and study model.^11^

In the present work, the insertion angles of the mini-screws were 30º, 60º, and 90º to the cortical bone surface. In the 60^o^ inserted mini-screws, the horizontal force generated 45% more strain on cortical bone than the oblique force. In contrast, 90^o^ and 30^o^ inserted mini-screws generated much lower strains on the cortical bone than the 60^o^ insertion angle.

Similar results were also obtained in other studies, which reported that the cortical and spongy bone stresses generated by force application to the mini-screws inserted at 90^o^ were less than those generated at both 30º and 60º.^12,17^ Similar results were also obtained from a study by Choi et al^18^ (2016) except for the 30º insertion angle. They found that the maximum von Mises stresses increased as the angle of insertion decreased. They detected that mini-screw insertion at 90º to the bone surface is preferable to minimize stresses concentrated on the supporting bone. Also, previous studiesfound that the stresses created on the cortical bone in 60º inserted mini-screws were more than those created with 30^o^ inserted mini-screws.^19^

On the other hand, other studies concluded that the mini-screw insertion angle significantly affected the primary stability with the best results gained with an insertion angle ranging from 60º to 70º.^20^ Zhao et al^21^ concluded that the oblique insertion angle of the mini-screws was preferred as it offered more primary stability compared to the vertical insertion. These differences from our findings might be attributed to differences in mini-screw design, geometry, length, diameter, and study model.

The force magnitude in the present study was 2 N, which is the optimum force that approximates the force applied to a mini-screw during orthodontic treatment as reported by previous studies, which concluded that orthodontists could apply loads to mini-screws immediately, as longer healing time did not offer more stability at forces up to 2 N.^14,22^

In the present work, for all different CBTs, we found that force direction had a minor effect on bone strains; however, the horizontal force generated slightly more strains on the cortical bone than oblique one for all the insertion angles, especially in the 60^o^ inserted mini-screws in which the horizontal force generated about 45% more strains on the cortical bone than the oblique one.

These results were consistent with those of Marimuthu et al,^23^ who suggested that force direction had a statistically insignificant effect on stress distribution in the bone surrounding mini-screws and concluded that the force direction has a negligible effect on mini-screw stability. Consistent with our findings, Lin et al^24^ reported that orthodontic force direction had an insignificant impact on cortical bone stresses. However, contrary to our results, Suzuki et al^25^ found that the amount of stress and area of distribution varied according to the direction of the applied load.

In the current work, the maximum von Mises stresses concentrated in the mini-screw and cortical bone in all the models were 33.68 and 14.43 MPa, respectively. Both values were much lower than the known yield stress of titanium (692 MPa) and cortical bone (200 MPa), respectively.^11^

The primary limitation of this study was that its results were derived using a model and thus might not be applicable to clinical situations. Other intraoral factors could not be simulated in the finite element model, such as insertion torque and factors related to the gingiva.

## Conclusion

- Increasing the CBT slightly reduces the exerted microstrain on bone.
- Inserting mini-screws at 60^o^ to the bone surface should be avoided as it generates much more strains on cortical bone than 90^o^ and 30^o^, especially when a force parallel to the bone surface is applied.
- Force direction affected bone strains; the horizontal force generated more strains on cortical bone than the oblique one. Also, stresses on mini-screws were significantly increased by the force direction except for vertical insertion.
- Titanium mini-screws and cortical bone had sufficient strength to withstand force magnitudes up to 2 N.

## Authors’ Contributions

ONA conceived and designed the work, collected data, contributed to data analysis, and wrote the manuscript. MSS and SMH contributed to work design, supervised the work, critically revised the article, and approved the version to be published.

## Acknowledgments

None.

## Funding

Self-funded.

## Competing Interests

The authors declare no conflict(s) of interest related to the publication of this work.

## Ethics approval

Not applicable.
